# Validation of a Complementary Food Frequency Questionnaire to assess infant nutrient intake

**DOI:** 10.1111/mcn.12879

**Published:** 2019-08-28

**Authors:** Amy L. Judd, Kathryn L. Beck, Christopher McKinlay, Ashleigh Jackson, Cathryn A. Conlon

**Affiliations:** ^1^ School of Sport, Exercise and Nutrition Massey University Auckland New Zealand; ^2^ Liggins Institute University of Auckland Auckland New Zealand

**Keywords:** dietary assessment, food frequency questionnaire, infant, nutrient, reliability, validation

## Abstract

Dietary assessment in infants is challenging but necessary to understand the relationship between nutrition and growth and development. Currently no simple, validated methods exist to assess nutrient intake in New Zealand (NZ) infants. Therefore, this study aimed to assess the relative validity and reproducibility of a Complementary Food Frequency Questionnaire (CFFQ) to determine nutrient intakes of NZ infants. Ninety‐five parent–infant pairs (infant age 10 ± 1 months) completed the CFFQ twice (CFFQ‐1 and CFFQ‐2), 4 weeks apart (to assess reproducibility). A 4‐day weighed food record (4dWFR) was collected between CFFQ administrations (to assess validity). Validity and reproducibility were assessed for intakes of energy and 18 nutrients using Bland–Altman analysis, Pearson's correlation coefficients, cross‐classification, and weighted Kappa (κ). The CFFQ showed acceptable validity: Nutrients from the CFFQ were comparable with the 4dWFR (bias, 9–28%), correlation between methods ranged from *r* = .18 (saturated fat) to *r* = .81 (iron; mean *r* = .52), 54% (mean) of participants were correctly classified (range 39% to 67%), and 7.1% (mean) misclassified into opposite tertiles (range 2.1% to 14.7%). There was acceptable agreement between the CFFQ and 4dWFR (κ = 0.20–0.60). The CFFQ showed good reproducibility: Correlations ranged from *r* = .34 (folate) to *r* = .80 (zinc); for 16 nutrients, >50% of participants were correctly classified, and for all nutrients, <10% of participants were grossly misclassified. All nutrients showed acceptable to good agreement (κ > 0.20). The CFFQ has acceptable relative validity and good reproducibility for assessing nutrient intake in NZ infants aged 9–12 months, making it a useful tool for use in future research.

**Key messages**

- Dietary assessment is challenging and necessary in infants, to understand the link between nutrition and growth and development.
- The CFFQ has acceptable relative validity and good reproducibility for assessing over 14 nutrients in NZ infants aged 9–12 months.
- The CFFQ could be used in future research to assess the complementary feeding period, where a simple tool is needed with little participant burden.
- Future research could assess the validity of the CFFQ within specific ethnic groups, within high risk infant groups, as well as further explore protocols to estimate breast milk intake.

## 1 INTRODUCTION

Rates of obesity are increasing in New Zealand (NZ), affecting one third of adults and one in eight children (Ministry of Health, 2017). Dietary intake in infancy, rapid early weight gain, and early cessation of breastfeeding have all been shown to influence adiposity in later life (Baird et al., 2005; Barker, Osmond, Forsen, Kajantie, & Eriksson, 2005; Günther, Buyken, & Kroke, 2007; Koletzko et al., 2009; Owen, Martin, Whincup, Smith, & Cook, 2005). Dietary intake in infants is highly transitional, changing at around 6 months of age from a diet consisting entirely of breast milk and/or infant formula to one containing a variety of foods by 12 months. Infants are nutritionally vulnerable and at an increased risk of impaired growth and development during this complementary feeding period as milk alone can no longer meet their dietary requirements (Ministry of Health, 2008; Pan American Health Organization, 2003).

There are concerns about the diets of NZ infants during the complementary feeding period including inadequate iron intake (Soh et al., 2002) and early cessation of exclusive breastfeeding (Morton et al., 2012). Appropriate assessment of dietary intake in young children is important in furthering our understanding of the links between diet and growth and development and could aid in evaluating the impact of any intervention strategies.

However, dietary assessment in children under 12 months of age is challenging. Infants' diets get progressively more diverse as they transition from milk to solids resulting in wide variation in daily dietary intake. Many infants rely on breast milk intake, which is difficult to quantify, and also consume very small amounts of food, making it difficult to estimate portion size and assess nutrient intake.

There are a lack of tools available to assess nutrient intake during the complementary period in infants, with debate regarding which method is most appropriate (Cade, Thompson, Burley, & Warm, 2002). Traditionally, food records have been used to assess dietary intake; however, they have a large participant burden, require multiple days of collection, and are time consuming. Therefore, in large population studies, food frequency questionnaires (FFQs) are commonly used, as they gather data without the time, burden, or expense that food records pose (Willett & Lenart, 2013). As dietary patterns and food preferences vary across time and population groups, FFQs need to be current and specific to the population of interest so their results can be interpreted with confidence (Cade et al., 2002; Metcalf, Swinburn, Scragg, & Dryson, 1997; Willett & Lenart, 2013). Newly developed or modified tools must be validated to ensure they are measuring what they claim to measure. There are very few FFQs that have been validated for assessment of nutrient intake in infants less than 12 months of age (Andersen, Lande, Arsky, & Trygg, 2003; Gondolf, Tetens, Hills, Michaelsen, & Trolle, 2012; Marriott et al., 2008; Marriott et al., 2009; Palacios et al., 2017) and none specific to NZ infants. Therefore, the purpose of this study was to determine the relative validity and reproducibility of a Complementary Food Frequency Questionnaire (CFFQ) to assess nutrient intake in NZ infants aged 9–12 months.

## 2 METHODS

### 2.1 Study population

Participants were the parent or main carer of an infant aged between 9 and 12 months and were recruited from around NZ, between 2016 and 2018 through social media and community groups. Participants were excluded if their baby had been born preterm (<37 weeks of gestation) or diagnosed with an illness or received medications that could impact on growth or dietary intake. Ethical approval was granted (NOR15/061). Participants provided written informed consent on behalf of themselves and their child.

### 2.2 CFFQ development and study design

Development of the semiquantitative CFFQ was informed by the Growing Up in NZ study (Morton et al., 2012) and national dietary guidelines for infants and toddlers (Ministry of Health, 2008). A list of foods relevant to infant dietary intake was created and prioritised based on the contextual and cultural appropriateness of each, before being assigned frequency and portion sizes to reflect typical intake in this age group. This included some commercial infant foods such as baby rice, rusks, puree fruit, and puree vegetables. The final CFFQ comprised of 49 food items under six food categories: milk and fluid, cereals and carbohydrates, dairy products, meat and protein, miscellaneous foods (including biscuits, confectionary, dried fruit, muesli bars, popcorn, cake, and hot chips), and vegetables and fruit. The CFFQ was reviewed by a registered dietitian and pretested in a small group of caregivers for comprehension (not part of this validation study). Nutrients of interest were total energy, protein, total fat, saturated fat, carbohydrate, fibre, vitamin E, folate, potassium, calcium, zinc, selenium, thiamin, riboflavin, niacin, vitamin C, vitamin B_12_, iodine, and iron.

A cross‐sectional study design was used to determine the validity and reproducibility of the semiquantitative CFFQ, which was delivered online through Survey Monkey. Sociodemographic information was collected with the first CFFQ administration (CFFQ‐1), including length of gestation, infant age, sex, ethnicity, number of siblings, and the most recent height, weight, and head circumference recorded in the infant's Well Child book. After completion of the CFFQ‐1, participants undertook a 4‐day weighed food record (4dWFR) to assess CFFQ validity. The CFFQ was then repeated (CFFQ‐2) 4 weeks after the first administration to assess reproducibility.

### 2.3 Data collection

The CFFQ asked about the quantity and frequency of foods eaten in the previous 4 days. Portion responses in the CFFQ were mainly in teaspoons and tablespoons to account for the very small portion sizes consumed in infants. The 4dWFR was conducted over four nonconsecutive days, including one weekend day. Participants in this cohort were to record intake on days they were with their children all day. All foods and fluids were weighed and recorded on a study form before being offered to the child, and then any leftover (or spilt) food or drink was reweighed and recorded. Instructions and examples of how the 4dWFR should be completed were provided. Caregivers were advised to account for all foods and beverages consumed, including night feeds. Participants were able to use their own household electronic scales or were sent a Tablefair White Electronic Scale.

Participants were asked to continue their child's typical food and fluid intake whilst completing the food record and to include the name and brand of the food or drink, as well as to record recipes and cooking method, where relevant. Participants were also asked to record the name, brand, and dose of any supplements their infant was taking. For formula‐fed infants, participants were instructed to weigh the dry powder and the volume of water used before reweighing the final quantity.

For both the 4dWFR and the CFFQ, breastfeeding duration and frequency was recorded and breast milk volume estimated using previously validated calculations based on milk composition during late lactation (7–20 months; Dewey, Finley, & Lonnerdal, 1984). The average intake of breast milk was estimated at 600 ml day^−1^ or 100 ml of milk for a feed lasting 10 min or longer. For feed times less than 10 min, a proportion of this (i.e., 10 ml min^−1^) was calculated (Dewey et al., 1984).

### 2.4 Data handling

Participants were contacted to obtain any missing or incomplete information. Participants who completed the full CFFQ‐1 and 4dWFR were included in the validity analysis, and those who completed both the CFFQ‐1 and CFFQ‐2 were included in the reproducibility analysis.

Data from both CFFQs and the 4dWFR were analysed for nutrient content in FoodWorks 9 (Xyris Software, 2018), utilising the NZ Food Composition Database (NZ, FOODfiles 2016). Food records and output nutrient data were checked for outliers. If specific foods or commercial infant food brands were not located in the NZ Food Composition Database, the product was created using the nutrition information panel of the product. For micronutrients not on the nutrition information panel, similar products were found in Australian or other NZ food databases, and the nutrition profile was based on these foods. All recipes were entered using cooked ingredients where possible. If a recipe was not available or a parent was unsure of the recipe (e.g., if the food had been prepared before the start of the study), a similar product was chosen. Only three infants were receiving a nutritional supplement, which was identified as a prebiotic and was not included in analysis. Two data sets were created for each 4dWFR and CFFQ: one with milk intake (breast milk and formula) and one without. Primary analyses included milk intake (breast milk and formula); secondary analyses excluded milk intake to explore the impact of the estimation of milk intake on CFFQ validity.

### 2.5 Statistical methods

Statistical analysis was undertaken using IBM SPSS 23.0 (Chicago, IL, 2015). Categorical data are reported as number and percentage and continuous data as mean and standard deviation (*SD*). The validity of the CFFQ‐1 compared with the 4dWFR was assessed by three methods. First, daily nutrient intakes derived from the CFFQ‐1 and 4dWFR were compared by Bland–Altman analysis (Bland & Altman, 1999). Because for most variables, differences increased with the mean, Bland–Altman analysis was performed on logarithmically transformed values, with bias represented by the ratio of geometric means. Limits of agreement of 95% are given for the ratio of geometric means. Second, the strength of linear association between the CFFQ‐1 and 4dWFR for nutrient intake was assessed by Pearson's correlation (Masson et al., 2003). Third, agreement between methods was assessed by cross‐tabulating the tertiles. Agreement was deemed adequate if >50% of participants were correctly classified into the same tertile and gross misclassification into opposite tertiles occurred for <10% of participants (Masson et al., 2003). The degree of agreement was further quantified with the weighted kappa (ĸ) statistic (Cohen, 1968). A weighting of 1 was used for participants classified into the same third by each dietary assessment method, 0.5 for adjacent thirds, and 0 for opposite thirds. Kappa ≥0.61 was deemed to indicate good agreement, between 0.60 and 0.20 acceptable agreement and <0.20 poor agreement (Lombard, Steyn, Charlton, & Senekal, 2015; Masson et al., 2003).

Reproducibility of the CFFQ was assessed by comparing nutrient intakes from the CFFQ‐1 and CFFQ‐2 using the same methods described above.

Primary analysis included milk intake and was performed with and without adjustment for energy intake. Energy‐adjusted nutrient intakes were calculated as the residuals from the regression of nutrient intake on energy (Willett et al., 1985). Secondary analysis explored the impact of excluding milk intake in nutrient estimates. For all tests, a *P* value <.05 was considered to represent a statistical significance.

Use of power calculations in methods comparison studies is controversial, but it is generally considered necessary to have at least 50 paired measurements for assessment of agreement (Bland & Altman, 1999; Cade et al., 2002). Therefore, we aimed to complete both the CFFQ‐1 and 4dWFR in a minimum of 50 and a maximum of 100 parent–child dyads.

## 3 RESULTS

### 3.1 Participant characteristics

Of 186 parent–child dyads who registered interest in the study, 95 completed both the CFFQ‐1 and 4dWFR (validity analysis) and 93 completed both the CFFQ‐1 and the CFFQ‐2 (reproducibility analysis). Participant characteristics are outlined in Table 1. In almost all cases, the participant was the mother of the child (98.8%). Infants had a mean (*SD*) age of 10 (1) months, half were female (50.6%), and the majority were of NZ European ethnicity (82.4%). Nearly three quarters of the infants had been breastfed at some stage (72.6%) and the mean (*SD*) age for starting solids was 5.5 (0.6) months. Most infants were offered solids before milk at the time of the study (70.5%).

**Table 1:** Participant characteristics

Characteristics	Study Participants^a^
	(*N* = 95)
	Mean ± *SD* or *n* (%)
Age of infant (month)	10 ± 1
Weight of infant (kg)	8.79 ± 1.20
Female infant^b^	43 (50.6)
Caregiver completing CFFQ^b^	
Mother	84 (98.8)
Father	1 (1.2)
Length of gestation (week)	39 ± 2
Ethnicity of infant^b^^,^ c	
NZEO	70 (82.4)
Asian	7 (8.2)
Māori	4 (4.7)
Pacific	1 (1.2)
Indian	2 (2.4)
Other	1 (1.2)
Family status^b^	
Only child	45 (52.9)
One sibling	28 (32.9)
Two or more siblings	12 (14.1)
Breastfed at any stage	69 (72.6)
Age started solids (months)	5.5 ± 0.6
Order of milk and solids	
Milk first	28 (29.5)
Solids first	67 (70.5)
Special diet^d^	19 (20.0)

Abbreviations: NZEO, New Zealand European; CFFQ, Complementary Food Frequency Questionnaire.

a Study participants are those who completed at least one CFFQ and the 4‐day weighed food record (93 participants completed the first and second CFFQ only).

b Ten participants missing data.

c Self‐reported *main* ethnicity. Other ethnicity is Czech.

d Special diet includes vegetarian or gluten‐free/dairy‐free diets.

### 3.2 Validity of the CFFQ

Energy and nutrient intakes for the CFFQ‐1 and 4dWFR are presented in Table 2. In unadjusted analysis, there was a positive bias (9–22%) for fat and saturated fat intake (meaning the CFFQ overestimated intake) and a negative bias (10–28%) for carbohydrate, fibre, folate, potassium, thiamin, riboflavin, niacin, and vitamin C intake (meaning the CFFQ underestimated intake; Table 2). Limits of agreement were approximately twofold above and below the geometric means. After adjustment for energy intake, there was no significant bias for nutrient intakes and limits of agreement decreased (Table 2).

**Table 2:** Validity of the Complementary Food Frequency Questionnaire among infants aged 9–12 months (*n* = 95)

Nutrients	CFFQ‐1	4dWFR	Bland–Altman		Correlation coefficient (*r*)		Correctly classified (%)		Grossly misclassified (%)		Weighted kappa statistic	
	Mean daily intake	Mean daily intake	RGM [95% LOA]									
			Raw	Adj	Raw	Adj	Raw	Adj	Raw	Adj	Raw	Adj
Energy (kJ)	3,306 ± 1047	3,295 ± 810	0.99 [0.54, 1.81]	—	.37†	—	44.2	—	10.5	—	0.25	—
Protein (g)	27.2 ± 10.3	26.6 ± 8.8	1.00 [0.52, 1.94]	1.03 [0.91, 1.17]	.51†	.70†	52.6	62.1	5.3	4.2	0.41	0.53
Fat (g)	37.1 ± 13.6	33.2 ± 8.7	1.09 [0.56, 2.11]*	0.98 [0.81, 1.19]	.35†	.43†	52.6	49.5	10.5	9.5	0.34	0.33
Saturated fat (g)	16.3 ± 6.2	13.5 ± 4.6	1.22 [0.43, 3.50]†	1.00 [0.79, 1.27]	.18	.36†	43.2	49.5	14.7	8.4	0.19	0.34
Carbohydrate (g)	83.6 ± 29.6	92.7 ± 31.2	0.90 [0.45, 1.78]†	1.00 [0.83, 1.20]	.41†	.36†	55.8	43.2	9.5	11.6	0.39	0.23
Fibre (g)	6.8 ± 3.6	9.3 ± 4.5	0.72 [0.24, 2.11]†	1.00 [0.80, 1.26]	.37†	.24*	46.3	39.0	9.5	12.6	0.29	0.18
Vitamin E (mg)	4.7 ± 2.4	4.8 ± 2.2	0.97 [0.38, 2.46]	0.98 [0.78, 1.23]	.47†	.39†	49.5	44.2	8.4	9.5	0.34	0.26
Folate (μg)	115.1 ± 54.2	137.8 ± 74.7	0.84 [0.29, 2.45]†	1.00 [0.83, 1.20]	.46†	.57†	50.5	60.0	12.6	6.3	0.30	0.48
Potassium (mg)	1,071 ± 434	1,257 ± 405	0.83 [0.40, 1.74]†	1.01 [0.83, 1.23]	.50†	.56†	45.3	64.2	8.4	7.4	0.29	0.52
Calcium (mg)	498 ± 246	478 ± 207	1.01 [0.51, 1.99]	1.03 [0.90, 1.18]	.73†	.78†	65.3	66.3	2.1	2.1	0.58	0.60
Zinc (mg)	5.1 ± 2.3	4.8 ± 1.9	1.03 [0.53, 2.01]	1.01 [0.88, 1.16]	.67†	.76†	60.0	62.1	4.2	3.2	0.50	0.54
Selenium (μg)	22.8 ± 8.1	23.8 ± 7.8	0.95 [0.44, 2.05]	1.00 [0.80, 1.24]	.27†	.36†	39.0	46.3	12.6	13.7	0.17	0.24
Thiamin (mg)	0.7 ± 0.5	0.8 ± 0.5	0.88 [0.33, 2.34]†	1.02 [0.87, 1.19]	.69†	.64†	57.9	51.6	3.2	6.3	0.49	0.38
Riboflavin (mg)	0.8 ± 0.4	0.9 ± 0.5	0.88 [0.41, 1.90]†	1.03 [0.90, 1.18]	.70†	.74†	54.7	63.2	4.2	3.2	0.44	0.55
Niacin (mg)	5.3 ± 3.0	6.1 ± 3.0	0.85 [0.41, 1.79]†	1.00 [0.85, 1.17]	.66†	.65†	57.9	51.6	3.2	5.3	0.49	0.40
Vitamin C (mg)	56.4 ± 29.6	64.6 ± 33.6	0.87 [0.34, 2.21]†	0.98 [0.77, 1.23]	.54†	.44†	60.0	60.0	5.3	12.6	0.49	0.41
Vitamin B_12_ (μg)	1.2 ± 0.8	1.3 ± 0.9	0.94 [0.30, 2.98]	1.02 [0.88, 1.19]	.71†	.68†	61.1	67.4	4.2	3.2	0.52	0.60
Iodine (μg)	49.4 ± 34.5	49.1 ± 29.6	0.94 [0.40, 2.21]	1.00 [0.87, 1.15]	.76†	.77†	60.0	63.2	4.2	1.1	0.51	0.58
Iron (mg)	5.5 ± 4.0	1.5 ± 0.6	0.95 [0.42, 2.14]	1.01 [0.88, 1.15]	.81†	.78†	67.4	68.4	2.1	3.2	0.61	0.61

Data are mean ± *SD*. In Bland–Altman analysis, bias is represented by the ratio of geometric means (RGM) with significant bias indicated by ^*^ *P* ≤ .05 or † *P* ≤ .01; 95% limits of agreement (LOA) for the RGM are given in square brackets. Milk (breast milk and formula) intake included in all analyses.

Abbreviations: Adj, adjusted for energy intake; CFFQ‐1, first Complementary Food Frequency Questionnaire; 4dWFR, 4‐day weighed food record.

* Significant correlation: *P* ≤ .05.

† Significant correlation: *P* ≤ .01.

In unadjusted analysis, there was significant correlation between the CFFQ‐1 and 4dWFR for all nutrients, except saturated fat (*P* = .07). Correlation coefficients ranged from .18 for saturated fat to .81 for iron, with a mean correlation coefficient of .52. After energy adjustment, intake estimates of all nutrients of the CFFQ‐1 and 4dWFR were significantly correlated, including saturated fat, with a mean correlation coefficient of .52.

In unadjusted analysis, at least 50% of infants were correctly classified into the same tertile for intake for 14 nutrients (protein, fat, carbohydrate, vitamin E, folate, calcium, zinc, thiamin, riboflavin, niacin, vitamin C, vitamin B_12_, iodine, and iron). Less than 10% were grossly misclassified (opposite tertile) for 14 nutrients. For individual nutrients, the percentage of participants correctly classified ranged from 39.0% for selenium to 67.4% for iron, with mean correct classification rate of 53.9% (Table 2). There was good agreement (>50% correctly classified) for all nutrients except energy (44.2% correct classification), saturated fat (43.2%), fibre (46.3%), potassium (45.3%), and selenium (39.0%). The percentage of infants grossly misclassified ranged from 2.1% for iron and calcium to 14.7% for saturated fat, with mean gross misclassification of 7.1%. For most nutrients, there was acceptable agreement between the CFFQ‐1 and 4dWFR with kappa values ranging from 0.20 to 0.60. There was good agreement for iron (κ > 0.60) but poor agreement for saturated fat and selenium (κ < 0.20).

When adjusted for energy intake, the CFFQ‐1 showed comparable if not better agreement with the 4dWFR with mean correct classification rate of 56.2% (range 39.0% for fibre to 68.4% for iron) and only 6.8% were grossly misclassified (range 1.1% for iodine to 13.7% for selenium). When adjusted for energy intake, all nutrients showed acceptable (κ = 0.20–0.60) or good agreement (iron κ > 0.60), except for fibre, for which there was poor agreement (κ < 0.20).

In secondary analysis, there was a negative bias (11–36%) for energy and all assessed nutrients except for protein, fat, saturated fat, and vitamin B_12_ (Table S1). The association between the methods was weakened when milk intake was excluded from analysis, ranging from .21 (vitamin E) to .60 (niacin) with an average correlation of .44. All nutrients were significantly correlated (*P* < .05). On average, half of participants (mean 50.3%) were correctly classified (range 42.1% for vitamin C to 60.0% for protein), whereas 9.2% of participants on average were grossly misclassified (range 6.3% for calcium and iron to 12.6% for energy and vitamin E). All nutrients showed acceptable agreement (κ = 0.20–0.60).

### 3.3 Reproducibility of the CFFQ

In unadjusted analysis, there was a positive bias (7–16%) on repeat administration of the CFFQ for protein, thiamin, riboflavin, vitamin B_12_, and iron (Table 3). Limits of agreement were approximately twofold above and below the geometric means. When adjusted for energy intake, there was no significant bias in nutrient intake, and limits of agreement decreased.

**Table 3:** Reproducibility of the Complementary Food Frequency Questionnaire among infants aged 9–12 months (*n* = 93)

Nutrients	CFFQ‐1	CFFQ‐2	Bland–Altman		Correlation coefficient (*r*)		Correctly classified (%)		Grossly misclassified (%)		Weighted kappa statistic	
	Mean daily intake	Mean daily intake	RGM [95% LOA]									
			Raw	Adj	Raw	Adj	Raw	Adj	Raw	Adj	Raw	Adj
Energy (kJ)	3,284 ± 1047	3,239 ± 905	1.00 [0.60, 1.67]	—	.62†	—	55.9	—	9.7	—	0.40	—
Protein (g)	27.0 ± 10.5	28.7 ± 11.3	1.07 [0.57, 2.02]*	1.00 [0.86, 1.16]	.66†	.68†	59.1	66.7	8.6	2.2	0.44	0.60
Fat (g)	36.9 ± 13.4	35.6 ± 9.9	0.99 [0.55, 1.77]	1.02 [0.86, 1.21]	.63†	.63†	40.9	57.0	6.5	4.3	0.25	0.46
Saturated fat (g)	16.2 ± 6.1	15.7 ± 4.6	0.99 [0.54, 1.82]	1.04 [0.88, 1.22]	.64†	.69†	47.3	71.0	8.6	5.4	0.30	0.61
Carbohydrate (g)	83.0 ± 29.8	81.3 ± 28.2	0.99 [0.55, 1.77]	1.00 [0.83, 1.19]	.63†	.57†	60.2	52.7	7.5	6.5	0.46	0.39
Fibre (g)	6.8 ± 3.6	7.1 ± 4.0	1.05 [0.41, 2.68]	0.96 [0.79, 1.17]	.63†	.57†	52.7	59.1	8.6	9.7	0.37	0.42
Vitamin E (mg)	4.7 ± 2.4	4.5 ± 2.0	0.98 [0.48, 2.02]	1.04 [0.88, 1.22]	.73†	.75†	59.1	59.1	4.3	5.4	0.49	0.48
Folate (μg)	114.9 ± 53.1	131.2 ± 90.8	1.08 [0.36, 3.26]	0.97 [0.80, 1.18]	.40†	.34†	57.0	48.4	7.5	7.5	0.43	0.33
Potassium (mg)	1,066 ± 439	1,087 ± 440	1.04 [0.53, 2.01]	1.00 [0.83, 1.20]	.58†	.53†	58.1	64.5	8.6	4.3	0.43	0.55
Calcium (mg)	491 ± 247	506 ± 251	1.03 [0.52, 2.04]	1.02 [0.88, 1.17]	.76†	.77†	66.7	63.4	4.3	4.3	0.57	0.54
Zinc (mg)	5.1 ± 2.3	5.1 ± 2.1	1.03 [0.57, 1.89]	1.02 [0.90, 1.17]	.76†	.80†	71.0	63.4	4.3	3.2	0.62	0.55
Selenium (μg)	22.8 ± 8.2	23.4 ± 10.5	1.01 [0.50, 2.05]	1.03 [0.86, 1.23]	.45†	.45†	54.8	47.3	6.5	6.5	0.42	0.33
Thiamin (mg)	0.7 ± 0.5	0.8 ± 0.6	1.16 [0.40, 3.32]†	1.01 [0.85, 1.19]	.67†	.58†	55.9	55.9	5.4	5.4	0.44	0.44
Riboflavin (mg)	0.8 ± 0.4	0.9 ± 0.5	1.13 [0.54, 2.38]†	1.01 [0.89, 1.16]	.75†	.72†	63.4	66.7	4.3	2.2	0.54	0.60
Niacin (mg)	5.3 ± 3.0	5.4 ± 3.2	1.02 [0.49, 2.10]	1.02 [0.86, 1.19]	.71†	.66†	63.4	50.5	8.6	6.5	0.49	0.37
Vitamin C (mg)	55.4 ± 28.7	53.6 ± 25.6	0.97 [0.42, 2.23]	1.08 [0.89, 1.30]	.65†	.51†	64.5	57.0	5.4	6.5	0.54	0.44
Vitamin B_12_ (μg)	1.2 ± 0.8	1.4 ± 0.9	1.12 [0.44, 2.88]*	1.00 [0.89, 1.13]	.82†	.69†	72.0	63.4	2.2	4.3	0.66	0.54
Iodine (μg)	49.2 ± 34.6	50.4 ± 36.0	1.04 [0.42, 2.6]	1.01 [0.87, 1.16]	.75†	.72†	59.1	64.5	2.2	3.2	0.51	0.56
Iron (mg)	5.5 ± 4.0	5.9 ± 4.1	1.13 [0.48, 2.67]†	0.99 [0.86, 1.13]	.80†	.74†	71.0	65.6	3.2	3.2	0.63	0.57

Data are mean ± *SD*. In Bland–Altman analysis, bias is represented by the ratio of geometric means (RGM) with significant bias indicated by ^*^ *P* ≤ .05 or ^†^ *P* ≤ .01; 95% limits of agreement (LOA) for the RGM are given in square brackets. Milk (breast milk and formula) intake included in all analyses.

Abbreviations: Adj, adjusted for energy intake; CFFQ‐1, first Complementary Food Frequency Questionnaire; CFFQ‐2; second Complementary Food Frequency Questionnaire.

* Significant correlation: *P* ≤ .05.

† Significant correlation: *P* ≤ .01.

Daily nutrient intakes of the CFFQ‐1 and CFFQ‐2 were significantly correlated, with coefficients ranging from .40 for folate to .82 for vitamin B_12_ (mean *r* = .67; all *P* < .01). When adjusted for energy intake, correlation coefficients were similar, ranging from .34 for folate to .80 for zinc (mean *r* = .63; all *P* < .01).

In unadjusted analysis, 59.6% of participants were classified, on average, in the same nutrient intake tertile on repeat administration of the CFFQ (range 40.9% for fat to 72.0% for vitamin B_12_; Table 3). For all nutrients, there was good agreement (>50% correctly classified) except for fat and saturated fat (40.9% and 47.3% correctly classified, respectively). On average, only 6.1% of participants were grossly misclassified on repeat administration of the CFFQ (range 2.2% for vitamin B_12_ and iodine to 9.7% for energy). When adjusted for energy intake, 59.8% of participants, on average, had corresponding classification (range 47.3% for selenium to 71.0% for saturated fat; Table 3). There was acceptable (κ = 0.20–0.60) or good agreement (κ > 0.60) for all nutrients between the two administrations of the CFFQ, with and without energy adjustment (Table 3).

In secondary analysis, there was a positive bias (11–26%) for protein, zinc, thiamin, riboflavin, and iron (Table S2). On average, 58.2% of participants were correctly classified into the same tertile (range 48.4% [niacin] to 65.6% [energy]). Similarly, all nutrients showed low levels of misclassification (<10% grossly misclassified) except niacin (10.8%). All nutrients showed acceptable agreement between the CFFQ‐1 and CFFQ‐2 (κ = 0.20–0.60).

## 4 DISCUSSION

The relative validity and reproducibility of the CFFQ to assess nutrient intakes of NZ infants aged 9 to 12 months were examined. Overall, the CFFQ showed acceptable to good validity for most nutrient intakes when compared with a 4dWFR, although minimum agreement criteria were not met for saturated fat, fibre, fat, and selenium. For energy, agreement was borderline but there was no bias, and limits of agreement were acceptable. The CFFQ had good reproducibility. Given the challenges of employing WFRs in large studies, the CFFQ appears to provide a practical alternative for semiquantitative assessment of nutrient intake in infants during the period of complementary feeding and may be useful in cohort studies to compare groups and assess the effect of interventions on early nutrition.

### 4.1 Validity

Energy intake appeared similar between the CFFQ and 4dWFR with only a 1% bias; however, there was significant variation between individuals; hence, overall agreement was borderline. Adjusting for energy intake may be useful in mitigating the effects of measurement error (Andersen et al., 2003) and improve the level of agreement for individual nutrients. When assessing groups such as in clinical studies, energy adjustment helps to highlight the quality of dietary intake. Energy adjustment for this reason can be advantageous in analyses of diet‐disease associations; however, interpretation of energy‐adjusted data is not straightforward and should be justified in clinical studies (Willett, Howe, & Kushi, 1997). More consistent results for all nutrients was found with energy adjustment. For the energy‐adjusted data, the CFFQ is able to adequately rank infants for most nutrients except fibre (κ < 0.20).

Although the CFFQ overestimated intake of fat, overall, there was a tendency to underestimate nutrient intake, including for carbohydrate, fibre, folate, potassium, thiamin, riboflavin, niacin, and vitamin C. In contrast, other studies in preschool children have generally shown that FFQs overestimate nutrient intake (Andersen et al., 2003; Bell, Golley, & Magarey, 2013; Gondolf et al., 2012; Livingstone & Robson, 2007; Marriott et al., 2008; Marriott et al., 2009; Palacios et al., 2017; Watson et al., 2015). These studies varied in methodology, nutrients assessed, reference methods, and timeframes. They also included children outside of the narrow age range in our study and so may not be directly comparable.

A likely contributor to the underestimation and overestimation of nutrients in FFQs is the difficulty in adequately estimating portion size, particularly in infants, as they typically taste food without eating the whole portion, making actual intake hard to quantify. FFQs are also limited in the amount of detail that can be obtained as the “frequency” and “amount” options are set. The CFFQ was designed to include measurements more closely related to the intake of infants (teaspoons and tablespoons), but portion size may still have been difficult to conceptualise.

Milk intake is a major contributor to an infant's energy and nutrient intake, typically accounting for a third or more of nutrient intake in infants less than 12 months of age. Breast milk intake has been handled differently in validation studies in young children (0–24 months): Either included in analysis (using estimates of volume; Andersen et al., 2003; Marriott et al., 2008; Marriott et al., 2009) or excluded from analysis (Gondolf et al., 2012; Watson et al., 2015), to our knowledge, only the study by Palacios et al. (2017) has compared the validity of FFQs with and without milk intake. Similar to this study, we found poorer agreement between methods when milk intake was excluded, which was not unexpected considering the large contribution of milk to an infant's diet. It is therefore important to include breast milk and/or formula in dietary assessment of children under 12 months of age, despite the limitations of milk volume estimation. More research is needed to overcome these limitations.

Correlation coefficients observed in our study (*r* = .52, range .18–.81) are compared favourably with other studies in infants and young children, particularly for vitamin C and E, riboflavin, calcium, potassium, iron, and zinc (Lovell, Bulloch, Wall, & Grant, 2017; Ortiz‐Andrellucchi et al., 2009). Correlation coefficients are affected by the agreement of the reference data, mode of administration, age, sex, and ethnicity of the study population (Preston, Palacios, Rodriguez, & Velez‐Rodriguez, 2011). We had a large sample size (*n* = 95), which may have improved correlations despite only 4 days of food records. We also had a narrow age range (infants 9–12 months), whereas other studies included younger infants (with less varied diets and improved associations; Marriott et al., 2008) and older infants (with more varied diets and likely reduced associations; Marriott et al., 2009).

There is no single best method for assessing validity of FFQs. Most studies rely on correlations to assess validity, despite correlations not measuring agreement between methods (Bland & Altman, 1986). Comparatively, cross‐classification gives a much clearer and undistorted picture of how well a FFQ performs (Cade et al., 2002) as FFQs are semiquantitative in nature. We assessed correlation for comparability with other studies and also compared methods (validity) and repeated administrations (reproducibility) using cross‐classification and Bland–Altman analysis. The agreement across tertiles between methods in our study (mean 53.9% [range 39%–67%] for correct classification) was higher than studies in infants and toddlers in the systematic review by Bell et al. (2013; average 36–38% correctly classified). However, the proportion of infants grossly misclassified (mean 7%, range 2%–15%) was slightly higher than in the review (3–5%).

The lack of tools currently available to assess nutrient intake in infants reflects the difficulties in dietary assessment in this age group. Although the CFFQ produces imperfect results, it still has utility for identifying infants with extremes of dietary intake and is an appropriate method for ranking infants according to nutrient intakes. The CFFQ may be useful for comparing population groups for these nutrients, particularly in large‐scale studies where a WFR would be difficult and burdensome for both the participant and researcher. It is possible that longer periods of recording may improve the agreement of these methods, but the increased risk of participant burden needs to be considered.

### 4.2 Reproducibility

The CFFQ showed good reproducibility, with all nutrients comparable between administrations apart from thiamin, riboflavin, vitamin B_12_, and iron, which were higher in the CFFQ‐2. This was not the case for a study in infants and toddlers which found no difference in the interpretation of results between administrations (Palacios et al., 2017). Reproducibility of a FFQ has been shown to be affected by several factors including ethnicity, sex, age, and education (Preston et al., 2011). All nutrients showed good cross‐classification (>50% correctly classified and <10% grossly misclassified) apart from fat and saturated fat. Overall, the CFFQ showed acceptable to good agreement for all nutrients (κ > 0.20). Similar results were seen with adjustments for energy intake.

A review of 227 validated FFQs by Cade et al. (2002) found only 47% of FFQs were tested for reproducibility. Assessing reproducibility is important as it determines how precise a tool is and impacts on the agreement between methods for individuals (Bland & Altman, 1999). Although repeated measures are not always made in method comparison studies (Cade et al., 2002), they are common elements of study design in clinical studies that assess change in variables over time. A strength of the current study is the simultaneous assessment of validity and reproducibility for the CFFQ. The CFFQ could be useful to assess dietary change in studies where multiple measures of nutrient intake over time are needed (particularly as infants have rapidly changing diets).

### 4.3 Strengths and limitations

One strength of the current study was using a WFR as the reference method, as it is the preferred and most precise method available for assessing dietary intake in infants (Luque et al., 2013). Alternative methods (collecting plasma or urine to test for dietary biomarkers or doubly labelled water technique) are expensive, invasive, and inefficient when looking at multiple nutrients. A limitation to this method is the participant burden and the researcher burden to process and analyse the information collected. Another limitation is the lack of standardisation of the scales used between participants (for those that used their own scale), which could impact the accuracy of the readings given, especially as infants may consume only small amounts of food. The CFFQ itself only offers a limited number of food and frequency options for participants to choose from, but in doing so reduces participant burden and is less labour intensive to analyse as a researcher.

We used 4 days of recall for both the CFFQ and WFR so we could effectively measure the same foods and nutrients and capture a similar number of days to assess nutrient intake as determined by other studies in infants (Boggio, Grossiord, Guyon, Fuchs, & Fantino, 1999; Lanigan, Wells, Lawson, & Lucas, 2001; Marriott et al., 2008; Marriott et al., 2009). Whereas shorter versions of FFQs may improve the validity and reproducibility (Cade et al., 2002), some nutrients require more than 4 days to accurately assess intake in children 6 to 24 months of age (Lanigan, Wells, Lawson, Cole, & Lucas, 2004; Nelson, Black, Morris, & Cole, 1989). It is likely that some of the nutrients with poorer agreement such as selenium, which are found in fewer foods, may have needed longer periods of recall to assess these adequately. However, recording food records for long periods is burdensome and could reduce the number of participants willing to complete the study (Livingstone & Robson, 2007). Further research could investigate whether a longer period of reporting in the CFFQ (e.g., intake over the last 2 weeks) would improve the agreement for these nutrients.

A potential limitation is that only 51% of those that registered interest in the study were able to complete it. Both the population group (infants) and the reference method (WFR) used in this study made it difficult for many parents to commit. However, considering the many challenges of assessing dietary intake in infants and the lack of tools already available, there were still a large number of participants (*n* = 95) able to complete the study. Previous studies in infants (<12 months) have only included 50 to 65 participants (Andersen et al., 2003; Marriott et al., 2008). For validity statistics, a minimum of 50 participants is needed, which might explain some of the differences in statistical results discussed above (Cade et al., 2002). Another limitation of this however was the self‐selected population, but in order to validate the CFFQ, motivated individuals are needed to complete it, so this does not necessarily make our results less valid.

Another limitation of our study is that few ethnic minorities were included, and it is possible that the diets of these infants are slightly different to that of European infants. For example, lower correlation between FFQs and reference methods have been observed in Pacific children (Metcalf et al., 2003). However, the study sample included participants from both urban and regional areas around NZ and thus is likely to be representative of the wider population.

Participants completed the CFFQ on two occasions 4 weeks apart and the 4dWFR immediately following the first administration of the CFFQ. It is possible that infants were introduced to a wider variety and quantity of food within this 4‐week period. However, if the two CFFQs were administered closer together, participants might remember the answers from the first administration, increasing the bias between methods. Furthermore, not all participants started the 4dWFR immediately after completing the CFFQ‐1 due to the infant being unwell or teething. If undertaking the 4dWFR when unwell or during teething, it may not have been a good representation of “usual” intake, due to changes in appetite. These discrepancies are difficult to quantify.

## 5 CONCLUSIONS

In conclusion, the CFFQ provided valid estimates of intake for 14 out of 19 nutrients in a group of NZ infants (9–12 months) compared with a 4dWFR (although less so for fibre and selenium). Although there was variability in assessment of individual energy intakes, there was no overall bias between the CFFQ and reference method for energy. Adjusting nutrient intake for energy resulted in a modest improvement in validity and may be preferred for assessments focusing on diet quality. The CFFQ demonstrated good reproducibility for all nutrients. The CFFQ is a valid tool for semiquantitative assessment of nutrient intake and diet quality and can be used for monitoring intake over time. The CFFQ may be particularly useful where a simple tool is needed with little participant burden to compare groups of infants who are progressing with complementary foods, including identification of unhealthy early dietary practices, and to investigate the impact of interventions aimed at improving early growth and nutrition.

## CONFLICTS OF INTEREST

The authors declare that they have no conflicts of interest.

## CONTRIBUTIONS

ALJ was responsible for recruitment, data collection, statistical analysis, formulation of results, and writing of the paper. AJ also contributed to recruitment and data collection. CC, KB, and CM developed the research protocol. CC obtained ethical approval and supervised the research process. KB and CM advised on statistical analysis and the formulation of results. All authors edited and approved the final paper.

## Supporting information

Table S1. Validity of the Complementary Food Frequency Questionnaire excluding milk intake among infants aged 9‐12 months (*n*=95).
Table S2. Reproducibility of the Complementary Food Frequency Questionnaire excluding milk intake among infants aged 9‐12 months (*n*=93).

Data S1. Supporting Information
