# Supplementary material for: Validation of a Complementary Food Frequency Questionnaire to assess infant nutrient intake
Source: Matern Child Nutr. 2019 Aug 28;16(1):e12879. doi: 10.1111/mcn.12879 (PMC7038889; doi:10.1111/mcn.12879)
Supplement: Supplementary file 1 — Table S1. Validity of the Complementary Food Frequency Questionnaire excluding milk intake among infants aged 9‐12 months (n=95). Table S2. Reproducibility of the Complementary Food Frequency Questionnaire excluding milk intake among infants aged 9‐12 months (n=93). [file MCN-16-e12879-s001.docx]

### Appendices: supplementary material

| **Supplementary Table 1 Validity of the Complementary Food Frequency Questionnaire excluding milk intake** **among infants aged 9-12 months (n=95).** | | | | | | | |
| --- | --- | --- | --- | --- | --- | --- | --- |
| ***Nutrients*** | ***CFFQ-1*** | ***4dWFR*** | ***Bland Altman*** |  |  |  |  |
|  | ***Mean daily intake*** | ***Mean daily intake*** | ***RGM [95% LOA]*** | ***Correlation Coefficient (r)*** | ***Correctly classified (%)*** | ***Grossly misclassified (%)*** | ***Weighted kappa statistic*** |
| **Energy (kJ)** | 1658 ± 774 | 1853 ± 720 | 0.87 [0.78, 0.95]† | 0.40† | 50.5 | 12.6 | 0.3 |
| **Protein (g)** | 18.3 ± 9.8 | 18.9 ± 8.4 | 0.93 [0.84, 1.03] | 0.56† | 60 | 8.4 | 0.45 |
| **Fat (g)** | 15.2 ± 8.8 | 14.2 ± 7.2 | 0.99 [0.86, 1.14] | 0.38† | 45.3 | 9.5 | 0.27 |
| **Saturated fat (g)** | 6.2 ± 4.0 | 5.7 ± 3.2 | 1.01 [0.86, 1.19] | 0.59† | 50.5 | 8.4 | 0.34 |
| **Carbohydrate (g)** | 43.2 ± 24.0 | 57.0 ± 28.3 | 0.74 [0.66, 0.82]† | 0.39† | 46.3 | 8.4 | 0.3 |
| **Fibre (g)** | 6.6 ± 3.6 | 8.5 ± 3.7 | 0.75 [0.67, 0.84]† | 0.41† | 44.2 | 10.5 | 0.25 |
| **Vitamin E (mg)** | 2.0 ± 1.3 | 2.3 ± 1.2 | 0.80 [0.70, 0.93]† | 0.21* | 49.5 | 12.6 | 0.29 |
| **Folate (µg)** | 96.2 ± 52.6 | 120.8 ± 75.2 | 0.80 [0.71, 0.90]† | 0.48† | 54.7 | 8.4 | 0.4 |
| **Potassium (mg)** | 674 ± 366 | 910 ± 384 | 0.72 [0.64, 0.80]† | 0.49† | 48.4 | 7.4 | 0.34 |
| **Calcium (mg)** | 190 ± 123 | 217 ± 120 | 0.82 [0.72, 0.94]† | 0.55† | 52.6 | 6.3 | 0.39 |
| **Zinc (mg)** | 2.5 ± 1.3 | 2.6 ± 1.2 | 0.89 [0.80, 1.00]* | 0.45† | 54.7 | 9.5 | 0.38 |
| **Selenium (µg)** | 11.2 ± 7.1 | 13.8 ± 7.5 | 0.78 [0.68, 0.89]† | 0.40† | 47.4 | 11.6 | 0.27 |
| **Thiamin (mg)** | 0.4 ± 0.4 | 0.6 ± 0.5 | 0.71 [0.61, 0.83]† | 0.55† | 51.6 | 11.6 | 0.32 |
| **Riboflavin (mg)** | 0.4 ± 0.2 | 0.6 ± 0.3 | 0.74 [0.65, 0.84]† | 0.42† | 54.7 | 9.5 | 0.38 |
| **Niacin (mg)** | 3.1 ± 1.9 | 4.3 ± 2.1 | 0.70 [0.64, 0.78]† | 0.60† | 57.9 | 8.4 | 0.43 |
| **Vitamin C (mg)** | 23.7 ± 19.2 | 36.5 ± 25.1 | 0.64 [0.55, 0.76]† | 0.35† | 42.1 | 9.5 | 0.25 |
| **Vitamin B_12_ (µg)** | 0.8 ± 0.5 | 0.9 ± 0.6 | 0.88 [0.73, 1.05] | 0.36† | 43.2 | 7.4 | 0.27 |
| **Iodine (µg)** | 12.8 ± 12.8 | 17.0 ± 10.1 | 0.64 [0.54, 0.75]† | 0.53† | 50.5 | 8.4 | 0.34 |
| **Iron (mg)** | 2.9 ± 1.5 | 3.4 ± 1.7 | 0.83 [0.75, 0.91]† | 0.59† | 51.6 | 6.3 | 0.38 |
| Data are mean ± SD. In Bland Altman analysis, bias is represented by the ratio of geometric means (RGM) with significant bias indicated by * p=<0.05 or † p=<0.01; 95% Limits of agreement (LOA) for the RGM are given in square brackets. Significant correlations are indicated by * p=<0.05 or † p=<0.01. CFFQ-1, first complementary food frequency questionnaire; 4dWFR, four-day weighed food record. Milk (breast milk and formula) intake excluded from analyses. | | | | | | | |

| **Supplementary Table 2**  **Reproducibility of the Complementary Food Frequency Questionnaire excluding milk intake among infants aged 9-12 months (n=93).** |
| --- |

| ***Nutrients*** | ***CFFQ-1*** | ***4dWFR*** | ***Bland Altman*** |  |  |  |  |
| --- | --- | --- | --- | --- | --- | --- | --- |
|  | ***Mean daily intake*** | ***Mean daily intake*** | ***RGM [95% LOA]*** | ***Correlation Coefficient (r)*** | ***Correctly classified (%)*** | ***Grossly misclassified (%)*** | ***Weighted kappa statistic*** |
|  |  |  |  |  |  |  |  |
| **Energy (kJ)** | 1638 ± 774 | 1765 ± 893 | 1.08 [1.00, 1.16] | 0.72† | 65.6 | 5.4 | 0.55 |
| **Protein (g)** | 18.2 ± 9.9 | 20.3 ± 11.3 | 1.13 [1.03, 1.25]* | 0.71† | 62.4 | 4.3 | 0.52 |
| **Fat (g)** | 15.0 ± 8.6 | 16.2 ± 9.8 | 1.12 [0.99, 1.26] | 0.63† | 58.1 | 9.7 | 0.41 |
| **Saturated fat (g)** | 6.1 ± 3.9 | 6.6 ± 4.5 | 1.14 [0.98, 1.32] | 0.73† | 52.7 | 5.4 | 0.40 |
| **Carbohydrate (g)** | 42.6 ± 24.0 | 45.2 ± 26.3 | 1.07 [0.98, 1.17] | 0.70† | 61.3 | 8.6 | 0.46 |
| **Fibre (g)** | 6.6 ± 3.6 | 7.0 ± 4.0 | 1.07 [0.97, 1.17] | 0.65† | 52.7 | 8.6 | 0.37 |
| **Vitamin E (mg)** | 1.9 ± 1.2 | 2.0 ± 1.2 | 1.03 [0.92, 1.15] | 0.53† | 60.2 | 5.4 | 0.49 |
| **Folate (µg)** | 96.0 ± 51.9 | 114.4 ± 92.4 | 1.12 [0.99, 1.27] | 0.41† | 60.2 | 7.5 | 0.46 |
| **Potassium (mg)** | 674 ± 369 | 706 ± 443 | 1.04 [0.95, 1.14] | 0.61† | 64.5 | 3.2 | 0.56 |
| **Calcium (mg)** | 186 ± 119 | 206 ± 162 | 1.06 [0.94, 1.19] | 0.73† | 60.2 | 1.1 | 0.54 |
| **Zinc (mg)** | 2.4 ± 1.3 | 2.7 ± 1.5 | 1.11 [1.00, 1.23]* | 0.66† | 62.4 | 4.3 | 0.53 |
| **Selenium (µg)** | 11.2 ± 7.2 | 13.3 ± 10.4 | 1.11 [0.98, 1.26] | 0.51† | 63.4 | 4.3 | 0.54 |
| **Thiamin (mg)** | 0.4 ± 0.4 | 0.5 ± 0.5 | 1.26 [1.08, 1.47]† | 0.62† | 62.4 | 6.5 | 0.50 |
| **Riboflavin (mg)** | 0.4 ± 0.2 | 0.5 ± 0.3 | 1.14 [1.01, 1.29]* | 0.59† | 58.1 | 6.5 | 0.45 |
| **Niacin (mg)** | 3.1 ± 1.8 | 3.4 ± 2.1 | 1.09 [0.98, 1.21] | 0.61† | 48.4 | 10.8 | 0.30 |
| **Vitamin C (mg)** | 22.9 ± 16.2 | 23.9 ± 18.7 | 1.00 [0.86, 1.16] | 0.44† | 50.5 | 8.6 | 0.34 |
| **Vitamin B_12_ (µg)** | 0.8 ± 0.5 | 0.9 ± 0.7 | 1.15 [0.99, 1.35] | 0.69† | 55.9 | 6.5 | 0.41 |
| **Iodine (µg)** | 13.0 ± 12.9 | 15.7 ± 19.0 | 1.09 [0.92, 1.30] | 0.67† | 57.0 | 3.2 | 0.48 |
| **Iron (mg)** | 2.9 ± 1.5 | 3.4 ± 2.5 | 1.15 [1.04, 1.28]† | 0.66† | 50.5 | 7.5 | 0.36 |
| Data are mean ± SD. In Bland Altman analysis, bias is represented by the ratio of geometric means (RGM) with significant bias indicated by * p=<0.05 or † p=<0.01; 95% Limits of agreement (LOA) for the RGM are given in square brackets. Significant correlations are indicated by * p=<0.05 or † p=<0.01. CFFQ-1, first complementary food frequency questionnaire; CFFQ-2, second complementary food frequency questionnaire. Milk (breast milk and formula) intake excluded from analyses. | | | | | | | |
