# Supplementary material for: Validation of a Complementary Food Frequency Questionnaire to assess infant nutrient intake
Source: Matern Child Nutr. 2019 Aug 28;16(1):e12879. doi: 10.1111/mcn.12879 (PMC7038889; doi:10.1111/mcn.12879)
Supplement: Supplementary file 2 — Data S1. Supporting Information [file MCN-16-e12879-s002.docx]

### Additional supplementary material: Complementary Food Frequency Questionnaire (CFFQ)
